# Helminth species specific expansion and increased TNF-alpha production of non-classical monocytes during active tuberculosis

**DOI:** 10.1371/journal.pntd.0009194

**Published:** 2021-03-02

**Authors:** Gezahegn Bewket, Amare Kiflie, Ebba Abate, Olle Stendahl, Thomas Schön, Robert Blomgran

**Affiliations:** 1 Department of Immunology and Molecular Biology, University of Gondar, Gondar, Ethiopia; 2 Ethiopian Public Health Institute, Addis Ababa, Ethiopia; 3 Department of Biomedical and Clinical Sciences, Linköping University, Sweden; 4 Department of Clinical Microbiology and Infectious Diseases, Kalmar County Hospital, Kalmar, Sweden; Universidade Federal de Minas Gerais, BRAZIL

## Abstract

Both *Mycobacterium tuberculosis* infection and helminths may affect innate immune mechanisms such as differential effects on monocytes towards the non-classical and intermediate subsets that favor bacterial persistence. Our aim, was to investigate helminth species specific effects on the frequency and functional activity of monocyte subsets in patients with active tuberculosis and healthy subjects. HIV-negative patients with active pulmonary tuberculosis (PTB) and community controls (CCs) in Gondar, Ethiopia were screened for helminth infection by stool microscopy. Flow cytometric analysis of peripheral blood mononuclear cells (PBMCs) and *ex vivo* stimulation with purified protein derivative (PPD) and helminth antigens were used to characterize the distribution of monocyte subsets and their function. A total of 74 PTB patients and 57 CCs with and without helminth infection were included. Non-classical monocytes were increased in PTB patients with Ascaris and hookworm infection but not in Schistosoma-infected patients. Ascaris had the strongest effect in increasing the frequency of non-classical monocytes in both PTB patients and CCs, whereas PTB without helminth infection did not affect the frequency of monocyte subsets. There was a helminth specific increase in the frequency of TNF-α producing non-classical monocytes in hookworm infected PTB patients, both with and without PPD-stimulation. Low-to-intermediate TB disease severity associated with increased frequency of non-classical monocytes only for helminth-positive PTB patients, and the frequency of TNF-α producing monocytes were significantly higher in intermediate and non-classical monocytes of helminth positive PTB patients with an intermediate disease score. Helminth infection affected the frequency of monocyte subsets and function both in TB patients and controls which was helminth species dependent in TB patients. The clinical role of this potential immunomodulatory effect needs further study and may affect the response and protection to tuberculosis in areas where helminth infections are endemic.

## Author summary

Monocytes are important cells for the early innate immune response and play an integral part during inflammation and infection. Classical monocytes, the dominant monocyte subset during homeostasis and health, have been linked to efficient TB protection. Intermediate or non-classical monocytes have instead been associated with uncontrolled inflammation (TNF-alpha), cell death, and poor protection against *Mycobacterium tuberculosis*. In areas endemic for intestinal helminths, the immunoregulatory effects of monocytes may affect development or progression of TB disease. The role of monocyte subsets during helminth/TB coinfection have not been studied. In Gondar, Ethiopia, we show that in patients with helminth infection, a helminth species dependent expansion of non-classical monocytes is triggered, where Ascaris and hookworm had the strongest effect in coinfected pulmonary TB-patients. The increase in non-classical monocytes was mainly detected in coinfected patients with a low-to-intermediate disease severity. Only coinfection with helminths and TB induced an increased TNF-alpha response in monocytes. Thus, we found a helminth species-specific dysregulation of monocyte subset distribution and functionality in coinfected TB-patients which could affect TB pathogenesis.

## Introduction

Tuberculosis (TB) and helminthiasis are two wide-spread infections with a considerable overlap, especially in tropical countries [1,2]. In TB endemic areas such as Ethiopia, helminth infection rates are higher in TB patients compared with household contacts and community controls [3–5]. Intestinal helminth infections have been described to have a negative impact on the clinical outcome to TB therapy. Higher number of disease-involved lung zones were found in helminth infected TB patients compared with helminth negative TB patients, at the end of TB treatment [6]. In active TB patients, asymptomatic helminth infections are strongly associated with an increased regulatory T cell and Th2 type immune response [5], and decreased frequencies of Th1, Th17 and CD8+ T cells [7], that could lead to impaired protective cellular immunity to *Mycobacterium tuberculosis* (Mtb) infection. Helminth infections can also increase immunoregulatory monocytes characterized by downregulation of proinflammatory cytokines [8]. However, there is a paucity of studies about the effect of chronic helminth infection on monocyte subsets and their biological function during TB.

Monocytes are heterogeneous phagocytic cells of the innate immune system that play a key role during the immune defense against Mtb. Based on the relative expression of CD14 and CD16 surface markers, human monocytes can be classified into three phenotypically distinct subsets: classical (CD14^++^CD16^-^) monocytes, intermediate (CD14^++^CD16^+^) monocytes and non-classical (CD14^+^CD16^++^) monocytes [9]. In a healthy individual, classical monocytes are the major subset making up 80–95% of all monocytes, while intermediate and non-classical monocytes comprises up to 2–8% and 2–11%, respectively [10].

Classical monocytes are proinflammatory, exhibit high phagocytic activity [11–13], and can produce reactive oxygen species [12]. Intermediate monocytes, also display high phagocytic activity but less so than classical monocytes [13], and have both pro-inflammatory [14,15] and anti-inflammatory characteristics [16]. Non-classical monocytes mainly patrol the inflamed tissues and are involved in wound healing and resolution of inflammation [17]. During early inflammation, mouse classical Ly6C^++^CD43^+^ monocytes differentiate into M1 (proinflammatory or classically activated) macrophages characterized by high Mtb killing capacity. Mouse non-classical Ly6C^+^CD43^++^ monocytes instead differentiate into M2 (anti-inflammatory or alternatively activated) macrophages with poor bactericidal activity [17,18]. M2 macrophages are highly involved in inflammation resolution and wound healing [19], and protection against helminthic infections [20].

During inflammatory diseases, non-classical and intermediate monocytes are highly expanded, and the normal proportion of monocyte subsets is changed [21]. In TB patients, intermediate and non-classical monocytes have been shown to increase [22–24]. Classical monocytes produce less TNF-α and high levels of IL-10, while intermediate and non-classical monocytes produce higher levels of TNF-α and less IL-10 in TB patients [22]. Mtb infected mice, receiving CD16^-^ monocytes exhibited high levels of IL-10 and TGF-β, and mice receiving CD16^+^ monocytes had higher levels of TNF-α in their lungs [25].

Helminth infection with filaria caused a significant expansion of non-classical monocytes [26]. The separate infections of helminths and TB mostly skew the expansion of monocytes towards a non-classical phenotype, which promotes mycobacterial persistence and intracellular multiplication [27]. Thus, we hypothesized that chronic helminth infection in TB may shift the balance of monocyte subsets towards the non-classical subset, which could impair innate immune mechanisms for control and protection against Mtb. Therefore, our aim was to investigate the distribution of monocyte subsets and their function in pulmonary TB patients (PTB) and healthy community controls (CCs) during chronic helminth infection.

## Materials and methods

### Ethical statement

Written informed consent was obtained from all study participants. The study obtained ethical clearance from Ethics Review Board of the University of Gondar, Ethiopia (O/V/P/RCS/05/1254/2016). All TB patients received treatment according to national guidelines [28] and helminth positive individuals were offered anti-helminth treatment as a part of the protocol.

### Study participants

After oral and written consent, newly diagnosed pulmonary tuberculosis patients were recruited consecutively during the period from 2016-07-25 to 2018-12-18 at the Directly Observed Treatment Short-course (DOTS) Clinics of University of Gondar Hospital, Gondar Health Center, Maraki and Azezo Health Centers in the Gondar area. The inclusion criteria were patients from 15–65 years old with a sputum smear positive result for acid fast bacilli (smear positive TB) or Xpert positive. The exclusion criteria were patients requiring hospital admission, HIV, pregnancy, clinical signs or medical treatment indicating any concomitant infectious diseases other than TB. None of the study participants had symptoms suggestive of active helminth infection during enrolment. All helminth positive patients or community controls were offered anti-helminth treatment, based on the Standard Treatment Guidelines for primary hospitals [29]. *S*. *mansoni* infected TB patients and CCs, were treated with praziquantel, with a dose calculated as 40 mg/kg in two divided doses, 4–6 hours apart. *Ascaris lumbricoides* and hookworm infected TB patients and CCs were treated with albendazole, with 400mg in a single dose.

Only QuantiFERON-TB Gold In-Tube (QFT) (Qiagen, Australia) negative healthy community controls (CCs) were included, from the same community as TB patients, at the blood bank of University of Gondar Hospital. CCs who were fulfilling the blood donation criteria and were QFT negative were also included from the nearby areas found around Gondar town. Moreover, all CCs had a TB-score value of ≤3 and did not show any clinical signs or symptoms suggestive of clinical tuberculosis.

### HIV-screening

Testing for HIV was done at the voluntary counseling and testing clinic and at the DOTS clinic as part of provider-initiated HIV testing and counseling program (PITC) according to the hospital routine with HIV 1/2 STAT-PAK Assay (Chembio Diagnostics systems Inc., USA), Uni-Gold Recombigen HIV-1/2 (Trinity Biotech, USA), and SD BIOLINE HIV-1/2 3.0 (Abbott, USA). HIV-positive patients were referred to the HIV clinics for further assessment and free antiretroviral treatment (ART) according to Ethiopian HIV/AIDS treatment guideline, and were excluded from the study.

### Clinical examination

A structured questionnaire was used to collect socio-demographic and clinical information. As previously described [30,31], the TB score which ranges from 0 to 13 points was assessed at baseline during initial inclusion. The TB score composed of signs and symptoms for TB (cough, haemoptysis, chest pain, dyspnea, night sweating, anemic conjunctivae, lung auscultation finding, tachycardia (≥100/min), temperature (≥37°C), body mass index (BMI) ≤18 kg/m2, BMI ≤16 kg/m2, mid-upper arm circumference (MUAC) ≤220 mm, and MUAC ≤200 mm), each having one point. TB disease severity was defined by TB score, and TB patients were classified in to three severity classes: severity class I (SCI: 0–5 points), severity class II (SCII: 6–7 points) and severity class III (SCIII: 8–13), as previously described [30].

### Laboratory investigations

The stool samples were collected once from each participant and the classification into helminth positive or negative were based on the examination of the stool samples by three stool examination techniques, using direct, Kato-Katz, and the formol-ether concentration technique [32,33]. Additionally, the egg count by Kato-Katz was used to determine the helminth burden according to the WHO’s guidelines for classification of helminth infection intensity (light, moderate, and heavy infection intensity; WHO/CTD/SIP/98.1)[34]. All stool examinations were performed by the same technician throughout the study. One in 10 slides were randomly selected and checked again blindly by a second microscopist for quality control. Acid fast bacilli (AFB) staining and evaluation was done using the direct method and a fluorescent microscope at baseline on spot-spot sputum samples according to national guideline by the same laboratory technician throughout the study. Sputum samples stored at -20°C for a maximum of 3 to 4 months were analyzed by Xpert, as previously described [35]. Xpert analysis was done for all patients including those who had been diagnosed with AFB, and PTB patients who were either Xpert positive or AFB positive were included in the study.

### Isolation of peripheral blood mononuclear cells (PBMCs) from whole blood

Heparinized venous blood (10 ml) was collected at DOTS clinics for TB patients, and for CCs at University of Gondar blood bank and from nearby villages to Gondar town, and transported within a maximum of two hours to the laboratory where PBMCs were directly isolated. The heparinized blood was diluted with equal volume of normal saline solution and carefully layered on the top of LymphoPrep density gradient solution (Serumwerk, Bernburg AG, Oslo, Norway), and then centrifuged at 800g for 30 minutes at 20°C. The resulting interphase ring consisting of a mixture of mononuclear cells was collected and then washed twice with PBS followed by centrifugation at 250g for 10 minutes. Finally, cells were resuspended in RPMI 1640 supplemented with 10% sterile heat-inactivated FBS and 1% antimycotic antibiotic solution (Sigma-Aldrich, Munich, Germany) before counting in a Bürker chamber with 0.4% trypan blue exclusion dye (Sigma-Aldrich, Munich, Germany) for detection of cell viability. All included donor samples had a cell viability above 75%, which was the cutoff point for the sample to be included for further analysis. Isolated PBMC cells were stored at -80°C using 10% dimethyl sulfoxide in fetal bovine serum, and used within 1–2 weeks for further analysis.

### Analysis of monocytes by flow cytometry

The cryopreserved PBMCs were thawed and washed, and included in the analysis if the cell viability was above 75% after thawing. PBMCs were stimulated with Mtb derived purified protein derivative (PPD; Statens Serum Institute, Copenhagen, Denmark) at a final concentration of 10 ug/ml, or media alone. Additionally, depending on the helminth status PBMCs were stimulated with 5 ug/ml of whole worm protein extracts from *Ascaris lumbricoides* from Allergen AB Thermo Fisher Scientific. After stimulation, cells were incubated at 37°C for a total of 6h. At 2h of stimulation, brefeldin A solution (10ug/ml) was added for the remaining 4h. Following the incubation period, PBMCs were treated with 5mM ethylenediaminetetraacetic acid (EDTA) for 15min at 37°C, washed and stained with the surface antibodies APC labeled anti-human HLADR (clone: LN3), PerCP-cyanine5.5 labeled anti-human CD14 (clone:61D3), and FITC labeled anti-human CD16 (clone: CB16), all from eBioscience. After staining of the monocyte subsets, PBMCs were fixed and permeabilized using BD Cytofix/Cytoperm and stained intracellularly according to the manufacture’s recommendation (BD Biosciences). Cells in Perm/Wash Buffer (BD Biosciences) were stained for intracellular cytokine expression using PE labeled anti-human TNFa (eBioscience, clone:MA611). Fluorescence minus one (FMO)-controls were used for gating purposes. Monocytes were first identified using their distinctive characteristics on forward/side scatter dot plot, and then HLADR positive cells were gated to exclude any CD16^+^HLADR^neg^ NK cells or other non-MHC expressing PBMCs as previously verified [36–38]. Monocyte subsets were then identified by CD14 and CD16 surface expression: classical monocytes (CD14^++^CD16^-^), intermediate monocytes (CD14^++^CD16^+^), and non-classical monocytes (CD14^+^CD16^++^). Starting with a lymphocyte gate based on lymphocytes forward/side scatter characteristics and thereafter proceeding with the same gating strategy used for monocytes showed that although some lymphocytes are HLADR^high^ they do not populate the subsequent CD14/CD16-monocyte gate. Additionally to the described sample treatment, one PBMCs aliquot of the thawed PBMCs without stimulation or *ex vivo* incubation was directly surface stained with the antibodies (against HLADR, CD14, and CD16), but without intracellular staining. This sample was used for reporting the frequencies of the monocyte subsets, whereas multi-stained samples including the anti-TNFa antibody (described above) was used to assess the TNF-production after the 6h *ex vivo* incubation. Flow cytometry data were collected on a FACS Calibur flow cytometer (BD Biosciences), using Cell Quest acquisition software and were analyzed using FlowJo 10.5.3 (TreeStar, USA).

### Statistical analysis

Continuous data are expressed as mean ± standard error of mean (SEM). Comparisons within CCs groups and within PTB groups was performed using one-way ANOVA followed by Tukey’s multiple comparison test. Comparisons between CCs groups and PTB groups after grouping into helminth negative and helminth positive was performed using two-way ANOVA followed by Tukey’s multiple comparison test. The main effect of disease severity between helminth negative and helminth positive PTB patients was analyzed using two-way ANOVA followed by Sidak’s multiple comparison test. We estimated that 16 patients in the helminth negative and positive groups respectively were needed to show an increase in non-classical monocytes from 10% to 25% with a power of 80% and an alpha of 0.05. Data analysis was performed using GraphPad Prism version 8.3.0 (GraphPad Software, San Diego, CA), and p values < 0.05 were considered statistically significant.

## Results

### Baseline characteristics of TB patients and healthy controls

**Fig 1:**
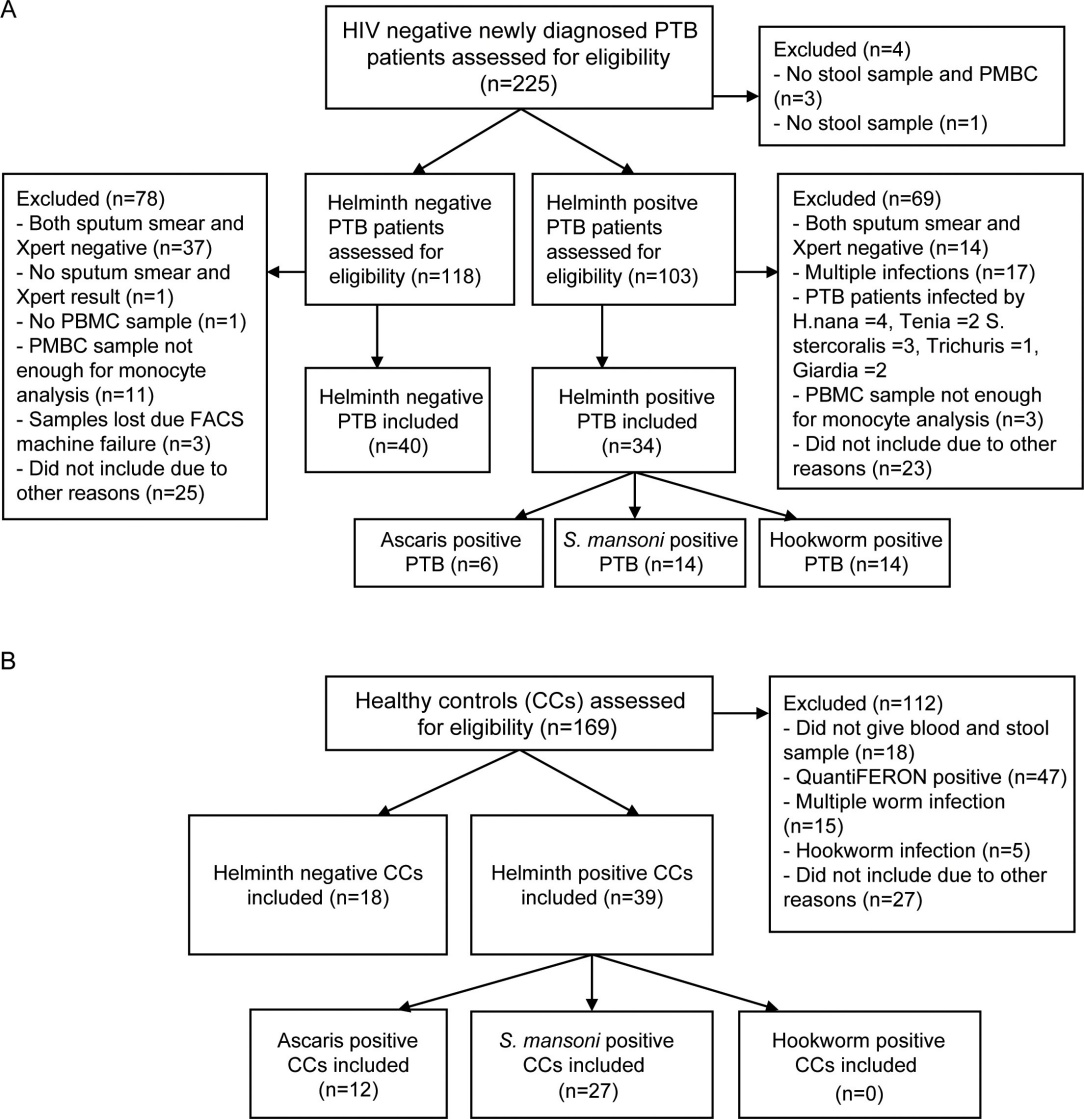
Flowchart of the inclusion process. (A) For pulmonary tuberculosis patients in the study conducted at four Directly Observed Treatment Short-Course Clinics of University of Gondar Hospital, Gondar Health Center, Maraki and Azezo Health Centers, Ethiopia. (B) For healthy community controls.

A total of 74 PTB patients with or without Ascaris, *S*. *mansoni* or hookworm infection, as well as 57 CCs with or without Ascaris or *S*. *mansoni* infection were included in the study (Fig 1). Hookworm positive CCs were not included in the study, since assessing impact of hookworm infection was not our initial aim while designing the study. There was no significant difference in median age between the PTB and CCs groups, and within each PTB group and CCs group. BMI was significantly higher in helminth negative CCs compared with Ascaris infected CCs (p < 0.05). No significant differences in BMI and TB score were observed between Ascaris, *S*. *mansoni*, hookworm infected and helminth negative PTB patients (Table 1). The helminth burden determined according to WHO’s guidelines for classification of helminth infection intensity showed that most CCs and PTB patients had the lowest infection intensity class, which is consistent with them being asymptomatic.

**Table 1:** Demographic characteristics of PTB patients and CCs and TB score value.

		Helm- /CCs	Ascaris+/CCs	*S*.*mansoni*+/CCs	P^C^	Helm-/PTB	Ascaris+/PTB	*S*.*mansoni*+/PTB	Hookworm+/PTB	P^TB^
N		18	12	27		40	6	14	14	
Sex (%)	M	66.7%	33.3%	63%	NA	61.5%	83.3%	78.6%	64.3%	
	F	33.3%	66.7%	37%	NA	38.5%	16.7%	21.4%	35.7%	
Age: median (R)		26.5 (20–32)	25.5 (17–58)	24 (18–57)	NS	22.5 (17–65)	22.5 (20–44)	27 (17–65)	25 (20–58)	NS
BMI: median (R)		22.1 (15.8–32)	19.1 (15.8–32)	20.3 (16.4–29.8)	P^CA^< 0.05	18.1 (14.4–23.2)	17.8 (15.4–20.2)	18.4 (14.9–24.6)	18.2 (15.5–19.3)	NS
TB score: median (R)					NA	6 (3–12)	6 (4–9)	7 (2–9)	6 (4–10)	NS
WHO Helm. Intensity (L/M/H)			(9/2/1)	(19/7/1)	NA		(4/2/0)	(10/2/2)	(14/0/0)	NA
%Class. Mo median (R)		69.5 (44.7–97.9)	51.3 (24.5–91.8)	62.1 (26.4–84.7)		81.6 (25.9–98.5)	53.5 (24.5–82.1)	82.8 (25.8–98.4)	62.3 (20.7–98.8)	
%Inter. Mo median (R)		13.3 (0.44–46.1)	11.9 (4.20–26.4)	11.6 (4.74–21.5)		13.3 (0.96–39.2)	18.8 (6.92–32.4)	9.94 (1.14–27.1)	13.9 (0.78–48.2)	
%Non-class. Mo median (R)		9.06 (0.53–35.2)	37.1 (3.76–57.7)	21.6 (3.19–63.9)		5.52 (0.58–54.9)	27.3 (1.34–68.2)	5.53 (0.46–47.1)	13.95 (0.46–69.0)	
%TNF+ Class. Mo (unstim.) median (R)		22.6 (5.65–71.6)	13.1 (1.28–63.3)	33.1 (3.51–69.9)		47.8 (7.90–88.5)	43.2 (28.0–56.4)	49.0 (12.1–81.6)	46.5 (6.72–87.5)	
%TNF+ Inter. Mo (unstim.) median (R)		15.0 (3.16–68.7)	10.4 (0–50.0)	19.7 (0–53.0)		26.6 (1.87–62.5)	32.8 (20.8–42.9)	42.2 (6.71–76.0)	34.3 (3.60–88.2)	
%TNF+ Non-class. Mo (unstim.) median (R)		4.86 (0–29.9)	3.15 (0–11.4)	3.26 (0–21.2)		4.97 (0–22.4)	6.71 (2.40–14.2)	5.96 (0–21.8)	9.74 (1.72–42.4)	

**Fig 2:**
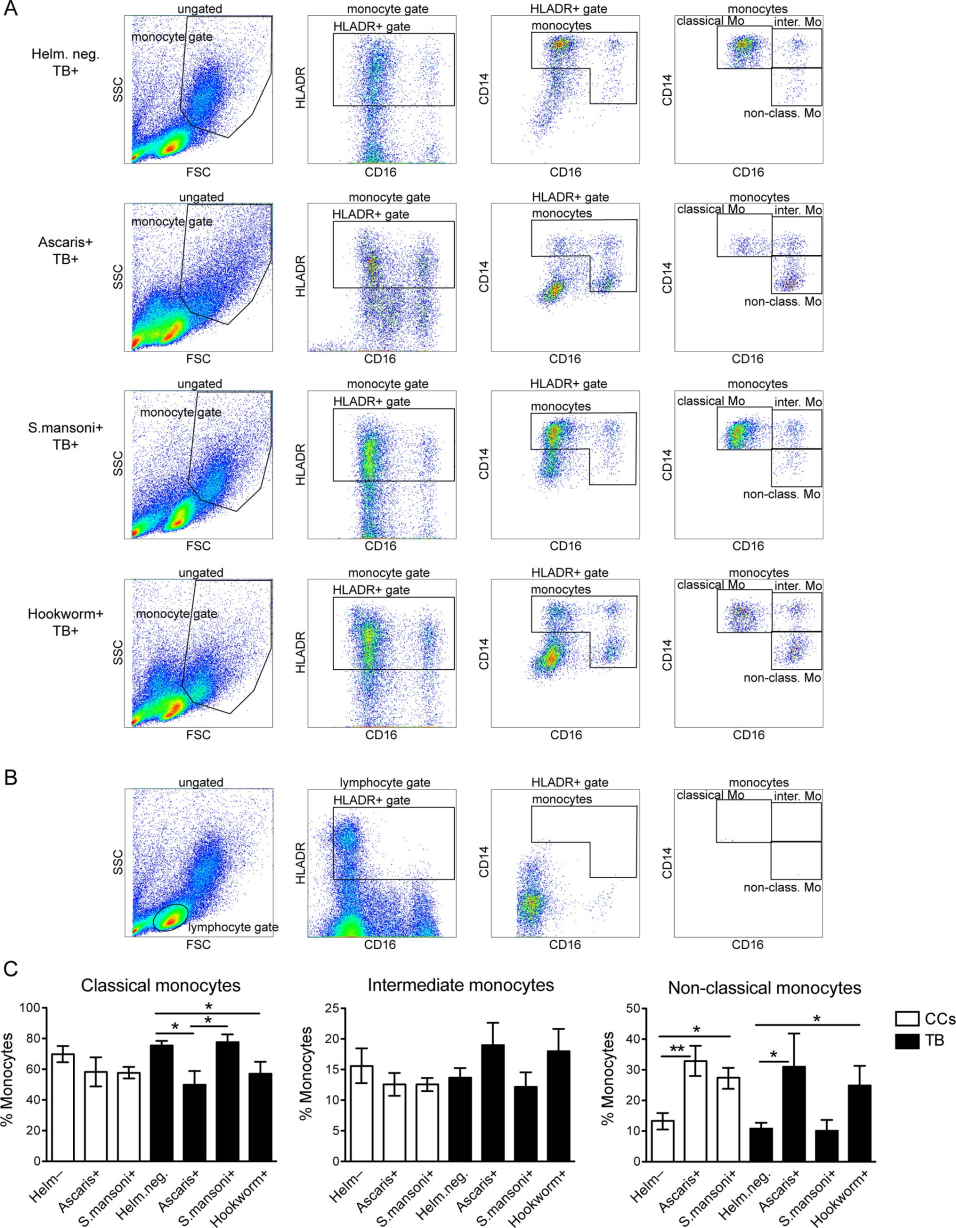
Monocyte subset distribution in QFT negative CCs and PTB patients according to their helminth species. Frequency of classical (CD14^++^CD16^-^), intermediate (CD14^++^CD16^+^), and non-classical (CD14^+^CD16^++^) monocytes in healthy community controls (CCs) and pulmonary tuberculosis patients (TB) before start of treatment. CCs and patients with or without (Helm-) the indicated asymptomatic helminth infection, and results were from PBMCs without *ex vivo* incubation. (A) Gating strategy for monocyte subsets including representative samples from patients in the TB group, starting with the characteristic forward/side scatter (FSC/SSC) gate of monocytes. Starting with the characteristic FSC/SSC gate of lymphocytes (from a sample in (A)) indicates that lymphocytes do not contaminate downstream monocyte gates (B). (C) Data is presented as the mean frequency ± SEM of monocytes within each monocyte subset. n = 18/12/27 for Helm-/Ascaris+/Schistosoma+ CCs, and n = 40/6/14/14 for Helm-/Ascaris+/Schistosoma+/hookworm+ PTB patients. Statistical analysis was made separately for CCs and PTB groups, using one-way ANOVA followed by Tukey’s multiple comparison test. Further, there was a significant decrease in classical monocytes in *S*. *mansoni*+/CCs versus *S*. *mansoni*+/PTB (p < 0.01) and increase in non-classical monocytes for *S*. *mansoni*+/CCs versus *S*. *mansoni*+/PTB (p < 0.01), when comparing CCs against PTB. *, p < 0.05; **, p < 0.01.

**Fig 3:**
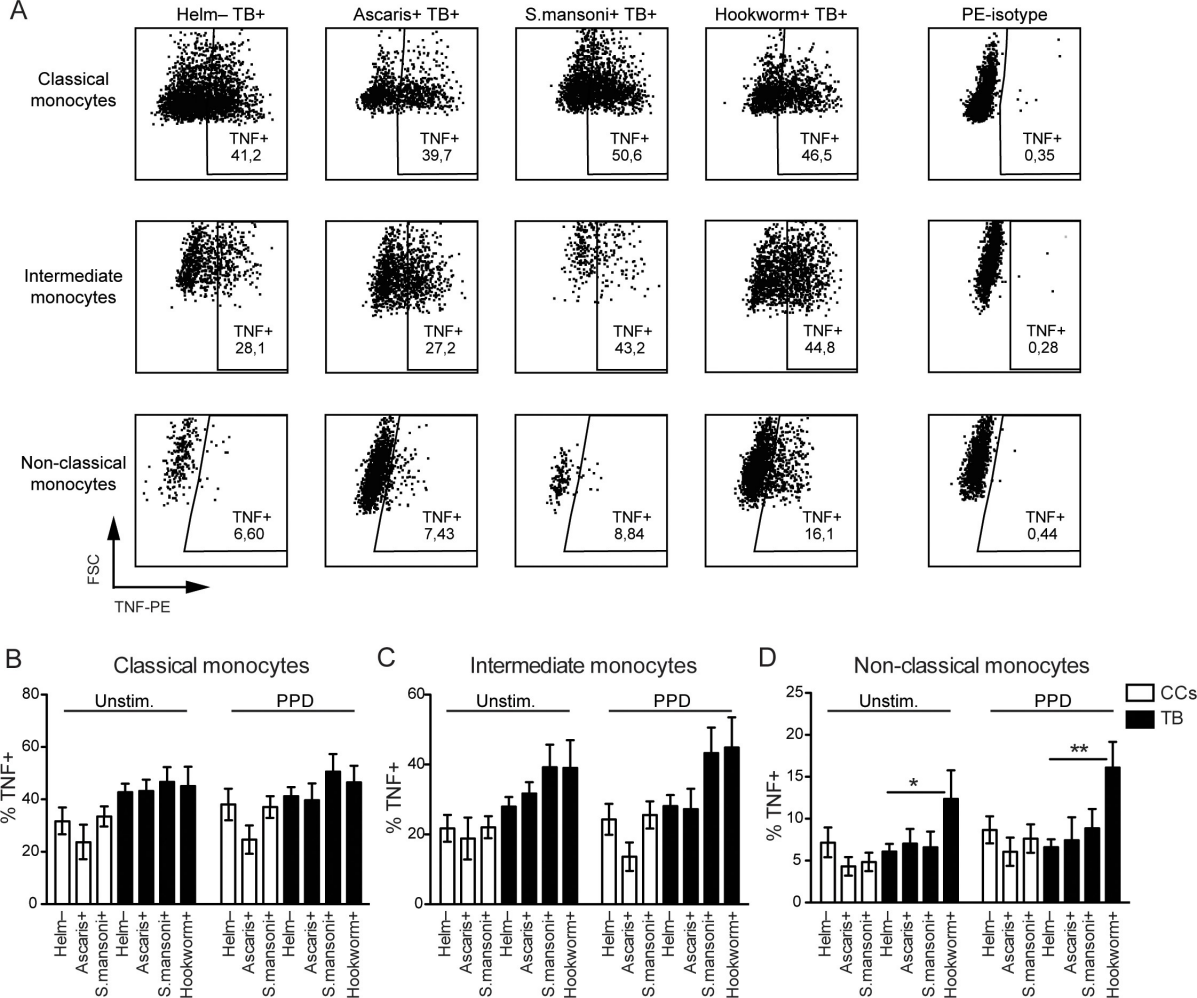
TNF-α producing monocyte subsets in QFT negative CCs and PTB patients according to helminth species. The frequency of monocytes within each subset of monocytes (classical, intermediate, and non-classical monocytes) in helminth negative (Helm-) and helminth positive (as indicated) after 6h *ex vivo* stimulation of PBMCs with medium (Unstim.) or PPD, where TNF positivity was analyzed by flow cytometry following intracellular staining with anti-human TNFα PE. (A) Intracellular PE-isotype stained PBMCs were used to set the TNF+ gates for the monocyte subsets, gated as in Fig 2A. Gating for PPD-stimulated PBMC from patients with TB is shown, and the numbers in gates are the mean values for each group. (B) Data is presented as the mean frequency ± SEM of TNF-α producing monocytes within each monocyte subset. n = 18/10/25 for Helm-/Ascaris+/Schistosoma+ CCs, and n = 35/6/14/14 for Helm-/Ascaris+/Schistosoma+/hookworm+ PTB patients. Statistical analysis was performed separately for CCs and PTB groups, using one-way ANOVA followed by Tukey’s multiple comparison test. Additionally to the differences within the PTB groups, there was a significant increase in % TNF+ intermediate monocytes in *S*. *mansoni*+/PTB compared to *S*. *mansoni*+/CCs, both in unstimulated and PPD-stimulated PBMCs (p < 0.05). *, p < 0.05; **, p < 0.01.

**Fig 4:**
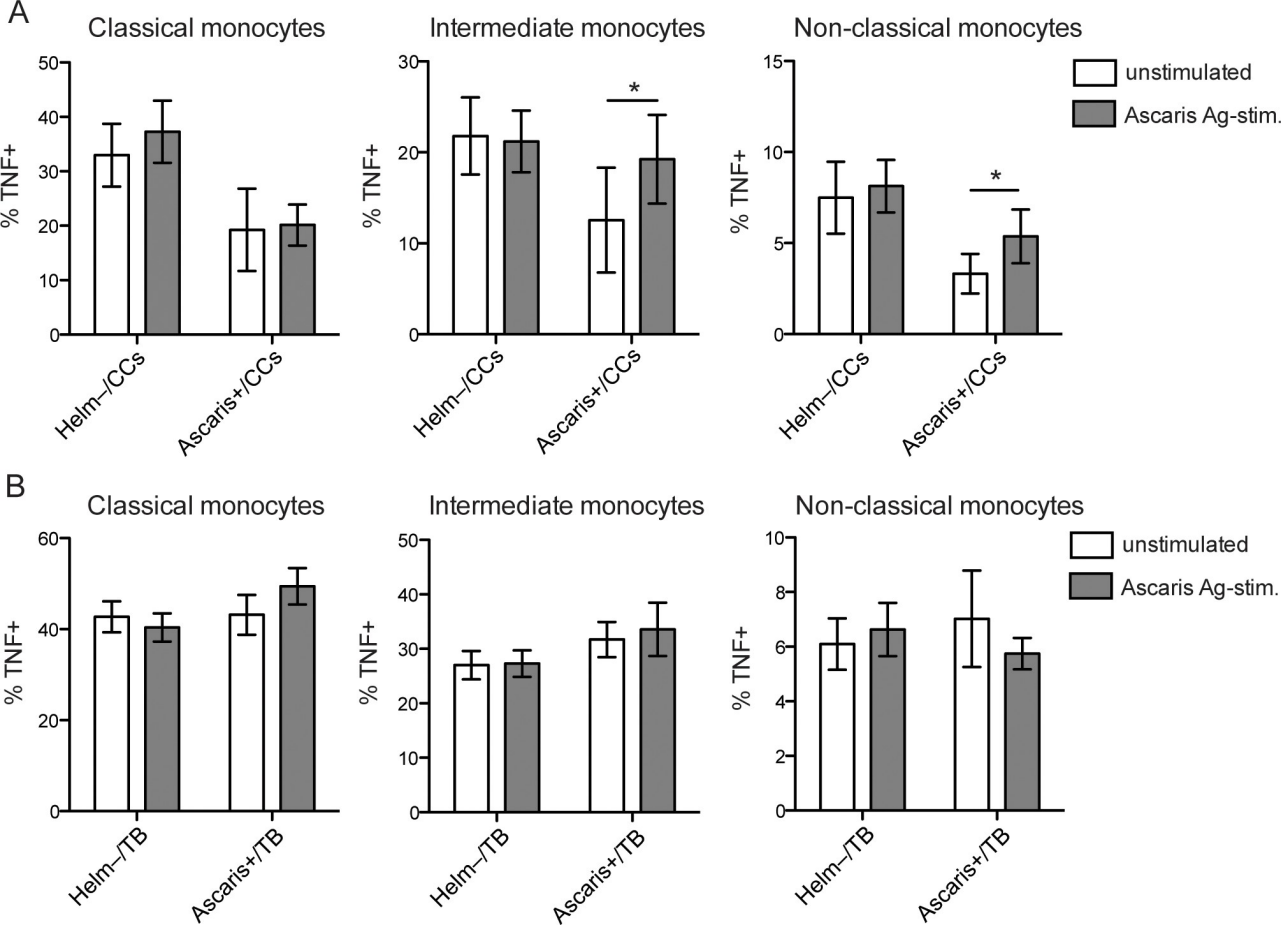
TNF-α producing monocytes in Ascaris positive CCs and PTB patients after Ascaris antigen stimulation. (A) QFT negative CCs with Ascaris infection and without helminth infection (Helm-). PBMCs were left unstimulated or stimulated with Ascaris antigen (Ascaris Ag) for 6h prior intracellular analysis of TNF-α in the monocyte subsets by flow cytometry. (B) Similar to (A), but for PTB patients with Ascaris infection and without helminth infection. Data is presented as the mean frequency ± SEM of TNF-α producing monocytes within each monocyte subset. p values calculated using paired t test for analyzing the recall response to Ascaris Ag in Ascaris positive CCs (n = 8), and in Ascaris positive PTB patients (n = 6). Ascaris negative CCs (n = 18) and Ascaris negative PTB patients (n = 34) are shown for reference and not included in the statistical analysis. *, p < 0.05.

**Fig 5:**
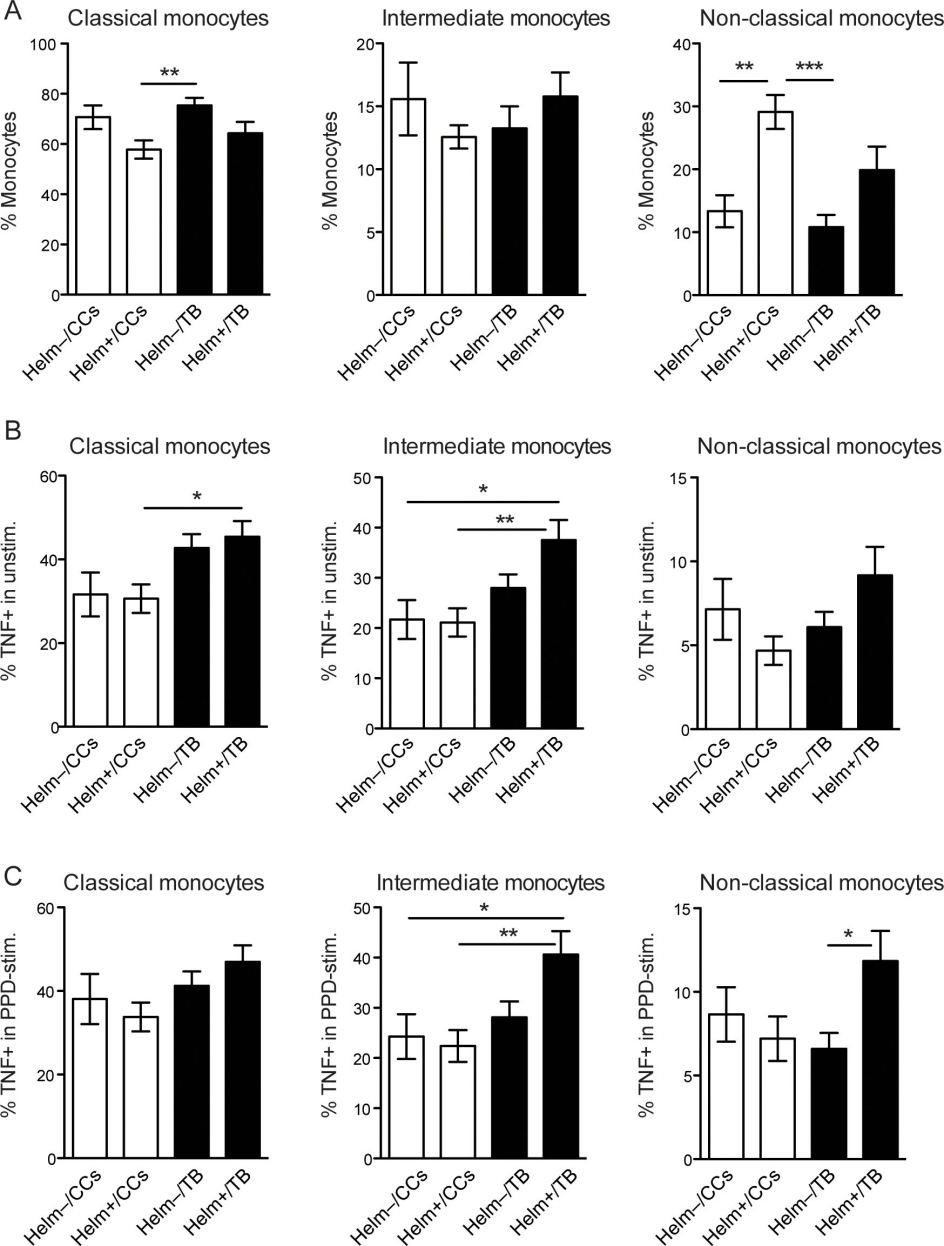
Monocyte subset re-distribution is helminth dependent and increased TNF-production only evident with helminth/PTB coinfection. (A) Analysis of PBMCs without *ex vivo* incubation. Frequency of classical, intermediate and non-classical monocyte subsets in QFT negative community controls (CCs) and PTB patients with (Helm+) and without (Helm-) helminth infection. (B) Frequency of TNF-α producing monocytes within each monocyte subset from (A) in PBMCs left unstimulated for 6h prior intracellular staining with anti-human TNFα PE and flow cytometric analysis of TNF+ monocytes. (C) Similar to (B) but with PPD stimulation of PBMCs for 6h and analysis of TNF+ monocytes within each monocyte subset. Data is presented as the mean frequency ± SEM of monocyte subsets and TNF-α producing monocytes within each subset. n = 18/35 for Helm-/Helm+ CCs, and n = 35/34 for Helm-/Helm+ for PTB. p values were calculated by two-way ANOVA followed by Tukey’s multiple comparison test. *, p < 0.05; **, p < 0.01.

**Fig 6:**
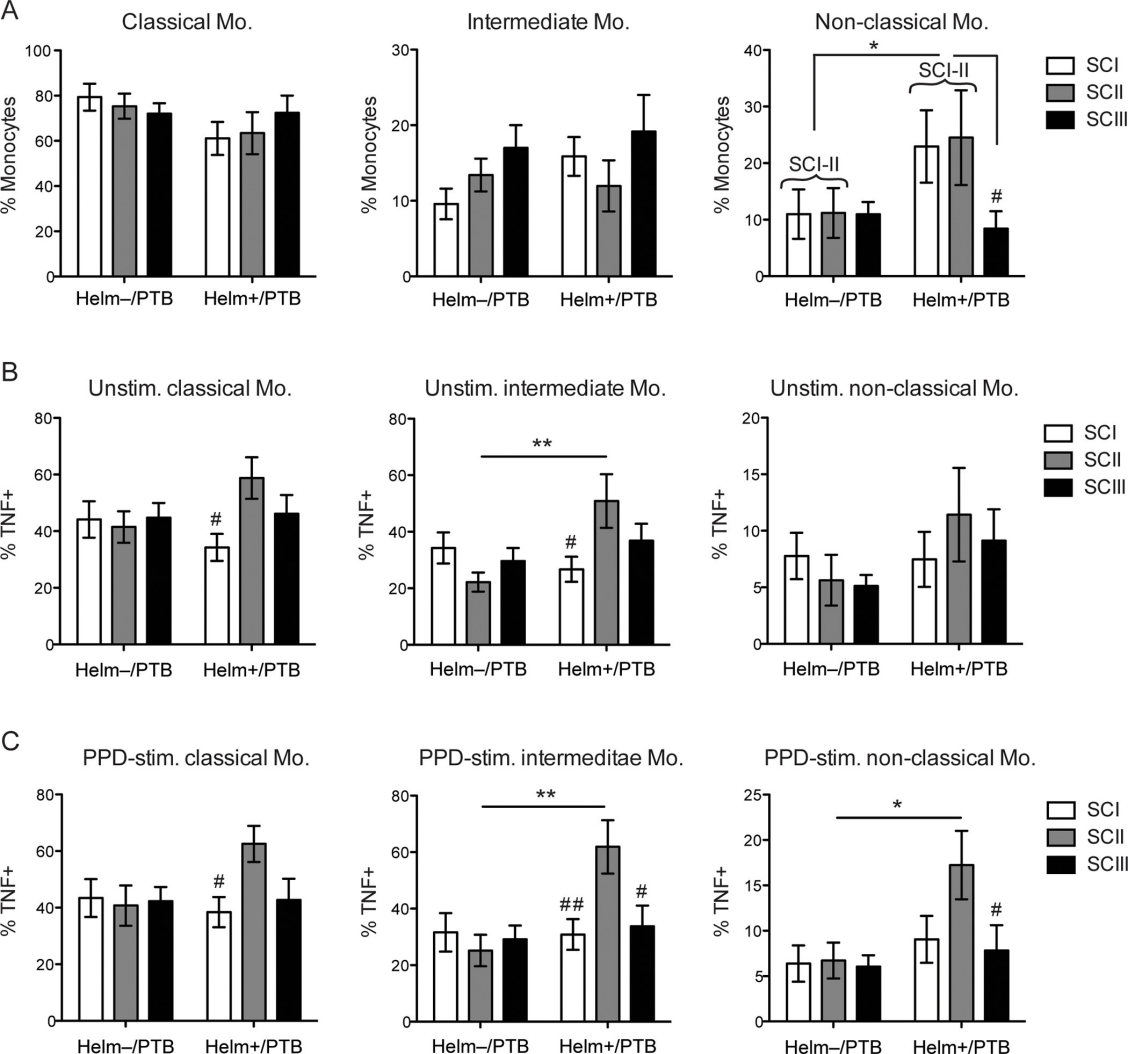
TB disease severity only correlate with alterations in monocyte subtype distribution and functionality during coinfection. (A) Analysis of PBMCs without *ex vivo* incubation. Frequency of classical, intermediate and non-classical monocyte subsets in pulmonary TB patients (PTB) based on their helminth status and disease severity using TBscore to classify patients into the three disease severity classes (SCI, SCII, SCIII). (B) Frequency of TNF-α producing classical, intermediate and non-classical monocytes in unstimulated PBMCs of PTB patients within the different disease severity classes. (C) Similar to (B) but for PBMCs stimulated with PPD for 6h. Data was from PTB patients with SCI (n = 13/13), SCII (9/10), and SCIII (n = 17/9), that were Helm-/Helm+, respectively. Data presented as the mean frequency ± SEM of monocyte subsets within each disease severity class of PTB patients, and the frequency of TNF-α producing monocytes within those. For non-classical monocytes in (A), brackets indicate the analysis when SCI and SCII (SCI-SCII) have been combined. p values were calculated by two-way ANOVA followed by Sidak’s multiple comparison test, and asterisks (*, **) show the main effect of disease severity between the helminth negative and helminth positive group. Additionally, the differences to SCII (or the combined SCI-II) within the group of helminth positive PTB patients are indicated by number sign (#, ##). */#, p < 0.05; **/##, p < 0.01.

Age: years; R: range; BMI: Body mass index in Kg/m^2^ (kilogram per meter square); CCs: community controls; PTB: Pulmonary tuberculosis; Helm-/CCs: Helminth negative CCs; Ascaris+/CCs: Ascaris positive CCs; *S*.*mansoni*+/CCs: *S*. *mansoni* positive CCs; Helm-/PTB: Helminth negative PTB; Ascaris+/PTB: Ascaris positive PTB; *S*.*mansoni*+/PTB: *S*. *mansoni* positive PTB; Hookworm+/PTB: Hookworm positive PTB; p values within CCs groups; P^CA^: p value of Helm-/CCs versus Ascaris+/CCs; P^TB^: p values within PTB groups; NA: not analyzed; NS: no p values were significant between groups. Only significant p values are displayed. WHO Helm. Intensity; Helminth infection intensity according WHO’s classification of egg burden into light/moderate/heavy (L/M/H), number of donors in each group; Median and range for classical monocytes (Class. Mo), intermediate monocytes (Inter. Mo), non-classical monocytes (Non-class. Mo), and % TNF positivity within these monocyte subsets left unstimulated (unstim.) are shown. Statistical comparisons for monocyte subsets and TNF-positivity are shown in Figs 2–6.

### The effect of helminths on the frequency of monocyte subsets in PTB patients is helminth species specific

To elucidate whether the effect of helminth infection on the frequency of monocyte subsets is species specific, we analyzed how Ascaris, *S*. *mansoni* and hookworm affected the distribution of monocyte subsets in both PTB patients and CCs. A significantly increased frequency of non-classical monocytes was observed in Ascaris+/PTB patients compared to Helm-/PTB patients (p < 0.05) (Fig 2). Ascaris infection also causes a significant elevation of non-classical monocytes in CCs (p < 0.01) (Fig 2). Similarly, non-classical monocytes were significantly increased in hookworm+/PTB patients compared to Helm-/PTB patients (p < 0.05) (Fig 2). In contrast to Ascaris and hookworm infection, *S*. *mansoni* infection did not alter the frequency of any monocyte subsets in PTB patients. However, in CCs, non-classical monocytes were significantly increased with *S*. *mansoni* infection (p < 0.05) (Fig 2), indicating that the effect of *S*. *mansoni* infection is different in PTB patients and CCs. The comparison between CCs and PTB groups supported this notion, showing a significant difference between *S*. *mansoni*+/CCs and *S*. *mansoni*+/PTB (p < 0.01), for both classical and non-classical monocytes.

From this analysis we conclude that helminth infections affected, the frequency of monocyte subsets in a species-dependent manner in PTB patients and that Ascaris induced an increase in non-classical monocytes both in the CC and PTB groups.

### Impact of helminth species on the functional activity of monocyte subsets in PTB patients and CCs

The frequency of TNF-α producing monocyte subsets was evaluated in PTB patients and CCs with and without helminth infection, to investigate whether helminth infection affects the functional activity of monocyte subsets (Fig 3). The frequency of TNF-α producing non-classical monocytes was significantly increased in hookworm-infected PTB patients compared to PTB patients without helminths (Helm-), in both unstimulated (p < 0.05) and PPD-stimulated PBMCs (p < 0.01). In contrast, Ascaris and *S*. *mansoni* infections did not significantly affect TNF-α production in any monocyte subset in neither PTB patients nor CCs (Fig 3). In the additional comparison between CCs and PTB, there was a significant difference in TNF-α producing intermediate monocytes between *S*. *mansoni*+/CCs and *S*. *mansoni*+/PTB for both unstimulated and PPD-stimulated PBMCs (p < 0.05).

### Impact of Ascaris infection on the functional activity of monocyte subsets after Ascaris antigen stimulation

As Ascaris had the strongest effect on the expansion of non-classical monocytes in PTB patients and CCs, Ascaris protein antigen was used to stimulate a recall response in PBMCs from PTB patients and CCs with Ascaris infection. In Ascaris+/CCs, Ascaris antigen caused a significant increase in TNF-α producing intermediate and non-classical monocytes (Fig 4A), whereas in the Ascaris+/PTB group with already increased or sustained TNF-α production the same effect was not evident (Fig 4B).

### Helminth infection affected the frequency of monocyte subsets and their function in PTB patients and CCs

Next, we combined all patients with helminth infection into one group, for PTB patients as Helm+/PTB and in CCs as Helm+/CCs, without differentiating between specific helminth species. This allowed comparison of Helm+ and Helm- groups as traditionally done in previous immunological studies on helminths, and also offered more reliable comparisons between the CCs and PTB groups. This analysis showed a significant increased frequency of non-classical monocytes in Helm+/CCs compared to Helm-/CCs (p < 0.01) (Fig 5A). Similarly, helminth infection in PTB patients caused a non-significant increase of non-classical monocytes. There were no significant differences between Helm-/CCs vs. Helm-/PTB nor Helm+/CCs vs. Helm+/PTB (Fig 5A). This suggests that TB as such does not affect the monocyte subset distribution, but that the observed effect on monocyte subset re-distribution is due to helminth infection.

The frequency of TNF-α producing monocyte subsets were also analyzed in these cohorts to elucidate whether general helminth infection affects the functional activity of monocyte subsets. We found that TNF-α producing classical monocytes were significantly increased in Helm+/PTB patients compared to Helm+/CCs (p < 0.05) in unstimulated PBMCs (Fig 5B). TNF-α producing intermediate monocytes were also increased in the Helm+/PTB group, compared to Helm+/CCs (p < 0.01), and to Helm-/CCs (p < 0.05), for both unstimulated and PPD-stimulated PBMCs (Fig 5B and 5C). In PTB patients, an increased frequency of TNF-α producing non-classical monocytes was observed in the Helm+/PTB group compared to the Helm-/PTB group after PPD-stimulation (p < 0.05) (Fig 5C). Thus, this analysis further illustrates that helminth infection drives monocyte subset re-distribution towards non-classical monocytes, independently of TB. Using TNF-α as a functional readout for monocytes, indicates that helminth infection alone does not affect monocyte function, whereas coinfection with helminths and TB increases TNF-α in both intermediate and non-classical monocytes.

### The impact of TB disease severity on monocyte subsets distribution and their function during helminth/TB coinfection

To assess whether TB disease severity, estimated by the TBscore subdivided in three severity classes (SCI-III) [30,39], affected monocyte subsets and their TNF-α production, we examined the frequency of each monocyte subset in helminth-negative and helminth-positive PTB patients. We observed that, Helm+/PTB patients with less severe TB disease (SCI and SCII combined) showed a significantly increased frequency of non-classical monocytes compared to the corresponding disease severity classes in Helm-/PTB patients (p < 0.05, n = 23 and n = 22 for SCI-II in Helm+/PTB and Helm-/PTB, respectively) (Fig 6A). Further, within the group of Helm+/PTB patients, those with less severe TB (SCI+SCII) showed significantly increased levels of non-classical monocytes compared to Helm+/PTB patients with severe disease (SCIII, n = 9). This indicates that disease severity affects the frequency of non-classical monocytes in helminth/TB coinfected individuals, but not by TB without helminth infection.

Monocytes from Helm+/PTB patients with SCII only had an overall increased frequency in TNF+ cells across all monocyte subsets compared to Helm-/PTB patients whereas there were no other differences observed for the other severity classes (Fig 6B and 6C).

## Discussion

Monocytes, take part in the early innate immune response and play an important role during inflammation and infection. The impact of helminth/TB coinfection on monocyte subsets have not been investigated in clinical studies previously. We show that Ascaris infection had the strongest effect on the expansion of non-classical monocytes in both PTB patients and CCs, followed by *S*. *mansoni* infection in CCs, and with hookworm infection in PTB patients. Our data show that helminth-induced monocyte subset alterations in PTB patients are species-specific in that Ascaris and hookworm infections, but not *S*. *mansoni*, significantly increased non-classical monocytes in PTB patients. This is in accordance with our previous study showing that helminths and their secreted antigens have a species-dependent impact on polarization of Mtb infected monocyte-derived macrophages [40].

Ascaris infection affects adaptive immunity through the induction of a strong Th2 type immune response characterized by an increased IL-4, IL-5 [41,42], IL-13 and IL-10 cytokine response [43]. However, studies on the effect of Ascaris on innate immune cells like monocytes are very scarce. *Ascaris suum* antigen was shown to inhibit *in vitro* differentiation of monocytes to M1 macrophage [44], and *Ascaris lumbricoides* cystatin to interfere with maturation of human monocyte-derived dendritic cells [45], in turn affecting T cell activation and interferon-gamma production [46]. Here for the first time, we show that *Ascaris lumbricoides* infection significantly expands non-classical monocytes, in both CCs and PTB patients, and additionally that hookworm infection increases non-classical monocytes in PTB patients. Similarly, a previous study demonstrated that regulatory monocytes were significantly increased in hookworm-infected individuals without TB [8].

The decreased frequencies of classical monocytes and increased expansion of non-classical monocytes, as shown in Ascaris-infected PTB patients and to a lesser extent also in hookworm-positive PTB patients, suggests that classical monocytes might be converted into non-classical monocytes as a result of helminth infection. The conversion from classical monocytes to non-classical monocytes may be due to an increased production of IL-10 and TGF-β, which is usually associated with helminth infections [5,47,48]. This is supported by *in vitro* IL-10 treatment of monocytes exhibiting enhanced CD16 expression [49]. *S*. *mansoni* infection significantly increased non-classical monocytes in CCs. However, in contrast to Ascaris and hookworm, *S*. *mansoni* did not affect the frequency of monocyte subsets in PTB patients. This shows that the effect of *S*. *mansoni* on the frequency of monocyte subsets is not the same for healthy controls and PTB patients. It has previously been shown that *S*. *mansoni* infection reduce the bacillary burden in the sputum of TB patients [50], and that *S*. *mansoni* antigen exposure of Mtb-infected human monocyte-derived macrophages reduce the intracellular Mtb burden with a concurrent decrease in IL-10 and maintained ability to activate Mtb-specific CD4 T cells [40]. Moreover, it was recently found that CD4 T cells of *Shistosoma mansoni* Mtb coinfected individuals (both with active or latent TB) have a plasticity in regard to their functional Th1 cytokine secretion where Th2 lineage positive CD4 T cells produced TNF, IFN-γ and IL-2 in response to Mtb-antigens [51]. Taken together, these studies show that *S*. *mansoni* coinfection in TB might induce TB protective Th1 type immune responses, which counteracts the expansion of non-classical monocytes.

It has been demonstrated that classical monocytes can control intracellular growth of Mtb, and that adoptive transfer of human classical monocytes to Mtb-infected SCID/Beige mice induces a higher lung migration rate and pulmonary infiltration of murine leukocytes leading to early Mtb infection control, compared to administration of CD16^+^ monocytes [25]. CD16^+^ monocytes are very permissive for intracellular mycobacterial growth and have an immune modulatory capacity [27] that can impair the proinflammatory environment and lead to chronic infection. CD16+ monocytes from TB patients were also impaired in their differentiation into dendritic cells [52], an important step in activating and generating Mtb-specific T cells. Thus, the expansion of non-classical monocytes, that we observe during coinfection with certain helminth species, may decrease the control of intracellular Mtb growth and contribute to enhanced susceptibility for TB.

Our result suggests that there was no TB specific impact on the frequency of monocytes, rather the observed perturbation of the frequency of monocyte subsets was due to helminth infections. This is in contrast to previous studies showing increased frequency of CD16+ monocytes [23] and both intermediate and non-classical monocytes in TB patients [53]. Focusing on newly diagnosed PTB that do not have other concomitant infections such as HIV or asymptomatic helminth infection, we do not observe TB specific effects on monocyte subset distribution, even when stratifying the patients according to their disease severity.

To assess whether the helminth-induced alteration of monocyte subsets correlates with their function, we measured the frequencies of TNF-α producing monocytes. Our data show that helminth infections in PTB patients modulate the cytokine production of monocyte subsets in a species-dependent way. For hookworm positive PTB patients there was both an expansion of non-classical monocytes along with an increased capacity for this subset to produce TNF-α, greatly increasing the total number of active cells. For Ascaris, there was a discrepancy in that non-classical monocytes were increased but without a concomitant increase in TNF-α production. This may be due to a lack of sensitivity in the assay to detect TNF-α as the number of non-classical monocytes were low but also that there may be stimuli specific subtypes and cytokine profiles of monocytes which are not detected by analyzing TNF-α only. It has previously been shown that TNF-α is produced by non-classical and intermediate monocytes from PTB patients, and that these CD16+ monocytes were more prone to undergo Mtb-induced cell death compared to their CD16- counterpart [22].

TB disease severity defined by TB score, showed that for helminth positive PTB patients non-classical monocytes were increased already in those with less severe disease (SCI+II). This could indicate one way by which helminths drive TB pathogenesis. However, no such effect was observed at any disease severity class among helminth negative PTB patients. This is in contrast with previous findings, where CD16^+^ monocytes expanded with increased TB disease severity and progression [23]. The difference might be explained by the method for TB disease severity classification, where chest X-ray classification [23] is cruder than TB score, which is the sum of signs and symptoms.

To focus on the specific effects of helminth infections, we selected only healthy controls without latent TB (QFT negative), and used three methods for stool examination of asymptomatic helminth infection in healthy controls and PTB patients. We were unable to make any correlations to helminth infection intensity as it was “light” (72% and 82% with “light” infection intensity among helminth positive CCs and PTB, respectively) according to WHO’s guidelines. Although the sample size is a limitation, we could analyze the data against a disease severity score revealing an important link between helminth infection, disease severity, and monocyte functionality. Our study is hypothesis driven and more studies are needed to confirm the results in the Ascaris positive group, for example a larger sample collection of Ascaris positive PTB patients would enable a comparison based on disease severity classes and TNF-production, and their response to Ascaris antigen restimulation. Another limitation was that a DUMP channel could not be included in the flow cytometry analysis, but this was adjusted for by carful gating strategies. In the PPD and Ascaris-antigen stimulation assays, our assay to measure specific TNF-α positive monocyte subsets by flow cytometry may not be sensitive enough to detect differences compared to total levels of TNF-α as measured by ELISA-based methods which on the other hand has the disadvantage of not being cell specific. Additionally, a potential limitation would be a difference in surface expression after the 6h incubation before analysis of TNF-α [54], but using the stimuli in our study, we did not find any effect on monocyte subset distribution after 6h (supporting information S1 Fig).

In summary, our study shows that helminth infections modulate monocyte subsets in a species- dependent manner in PTB patients. These results may have clinical implications in areas where both helminths and TB are endemic. The fact that helminths have different and species-dependent effects on TB-immunity should be considered when combating TB. The differential effect of helminths on monocyte subsets needs to be further explored, and large scale immunological and clinical studies need to be conducted to fully elucidate the impact of helminth species-specific effects on the immune modulation of monocyte subsets and their clinical impact in TB.

## Supporting information

S1 Fig No stimulation effect of Ascaris antigen or PPD on monocyte subset distribution in PBMCs. PBMCs from helminth negative pulmonary TB (PTB) patients (A) and Ascaris positive PTB patients (B) were either stained extracellularly (HLADR, CD14 and CD16) directly after thawing the PBMCs (unincubated), or incubated 6h *ex vivo* without (unstimulated) or with stimulation by Ascaris antigen (Ascaris-Ag) or PPD before being stained. Gating of the monocyte subsets was done as shown in Fig 2 in the main manuscript, and data presented as the mean frequency ± SEM of monocytes within each monocyte subset. All available data for unincubated and 6h incubated PBMCs for each respective group in A and B are shown.
(TIF)
