# Supplementary figures and images for: Helminth species specific expansion and increased TNF-alpha production of non-classical monocytes during active tuberculosis
Source: PLoS Negl Trop Dis. 2021 Mar 2;15(3):e0009194. doi: 10.1371/journal.pntd.0009194 (PMC7954301; doi:10.1371/journal.pntd.0009194)

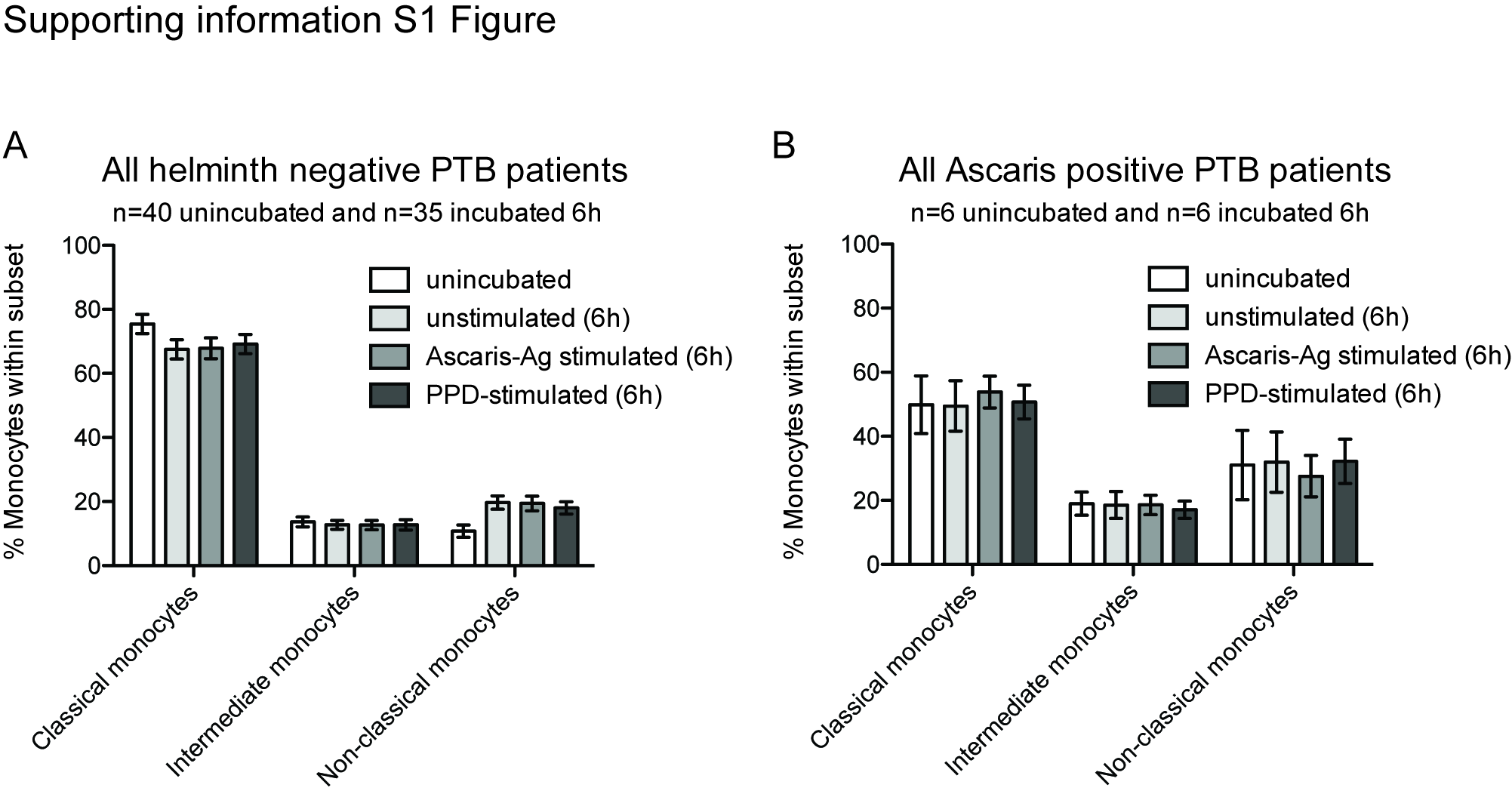

Supplement: S1 Fig — PBMCs from helminth negative pulmonary TB (PTB) patients (A) and Ascaris positive PTB patients (B) were either stained extracellularly (HLADR, CD14 and CD16) directly after thawing the PBMCs (unincubated), or incubated 6h ex vivo without (unstimulated) or with stimulation by Ascaris antigen (Ascaris-Ag) or PPD before being stained. Gating of the monocyte subsets was done as shown in Fig 2 in the main manuscript, and data presented as the mean frequency ± SEM of monocytes within each monocyte subset. All available data for unincubated and 6h incubated PBMCs for each respective group in A and B are shown. (TIF) [file pntd.0009194.s001.tif]
